# Effect of* Dangguibohyul-Tang*, a Mixed Extract of* Astragalus membranaceus* and* Angelica sinensis*, on Allergic and Inflammatory Skin Reaction Compared with Single Extracts of* Astragalus membranaceus* or* Angelica sinensis*

**DOI:** 10.1155/2016/5936354

**Published:** 2016-03-08

**Authors:** You Yeon Choi, Mi Hye Kim, Jongki Hong, Kyuseok Kim, Woong Mo Yang

**Affiliations:** ^1^Department of Convergence Korean Medical Science, College of Korean Medicine, Kyung Hee University, Seoul 02447, Republic of Korea; ^2^College of Pharmacy, Kyung Hee University, Seoul 02447, Republic of Korea; ^3^Department of Ophthalmology, Otorhinolaryngology and Dermatology of Korean Medicine, College of Korean Medicine, Kyung Hee University, Seoul 02447, Republic of Korea

## Abstract

*Dangguibohyul-tang* (DBT), herbal formula composed of* Astragalus membranaceus* (AM) and* Angelica sinensis* (AS) at a ratio of 5 : 1, has been used for the treatment of various skin diseases in traditional medicine. We investigated the effect of DBT on allergic and inflammatory skin reaction in atopic dermatitis-like model compared to the single extract of AM or AS. DBT treatment showed the remission of clinical symptoms, including decreased skin thickness and scratching behavior, the total serum IgE level, and the number of mast cells compared to DNCB group as well as the single extract of AM- or AS-treated group. Levels of cytokines (IL-4, IL-6, IFN-*γ*, TNF-*α*, and IL-1*β*) and inflammatory mediators (NF-*κ*B, phospho-I*κ*B*α*, and phospho-MAPKs) were significantly decreased in AM, AS, and DBT groups. These results demonstrated that AM, AS, and DBT may have the therapeutic property on atopic dermatitis by inhibition of allergic and inflammatory mediators and DBT formula; a mixed extract of AM and AS based on the herb pairs theory especially might be more effective on antiallergic reaction as compared with the single extract of AM or AS.

## 1. Introduction

Atopic dermatitis (AD) is one of the most common chronic and recurrent inflammatory skin diseases which affect environmental, genetic, immunologic, and biochemical factors [1]. The pathogenesis of AD has been known to be caused by T helper (Th) 1/2 dysregulation and skin barrier disorder [2, 3]. In addition, mast cells (MCs) in allergic diseases including AD have shown playing a crucial role in the secretion of histamine, leukotrienes, prostaglandin D2, proteolytic enzymes, and several cytokines including interleukin- (IL-) 1*β*, IL-4, IL-6, tumor necrosis factor- (TNF-) *α*, and interferon- (IFN-) *γ* [4].

In conventional medicine, most clinicians mainly focus on the regulation of T cell inflammation with corticosteroids, antihistamines, or immunosuppressive agents [5]. However, long-term uses of these agents can induce serious side effects such as facial edema, skin atrophy, striae distensae, and perioral dermatitis [6]. Therefore, a wide variety of plant-derived medicines with fewer side effects have been investigated as potential alternatives for allergic skin diseases instead of conventional therapy [7, 8].

Many studies have reported that natural products and their compounds inhibit the development of allergic skin diseases.* Dangguibohyul-tang* (DBT; herbal decoction), which combines simply with two herbs,* Astragalus membranaceus* (AM) and* Angelica sinensis* (AS), is widely used herbal formulas for the treatment of hematopoietic function, menopausal symptoms, and immune responses [9–11]. A recent pharmacological study indicated that DBT reduces inflammatory symptoms in AD-like mice [12]. Also, Dang-Gui-Yin-Zi, a similar herbal formula containing AM and AS, is commonly used for treating atopic dermatitis in clinical practice [13]. Additionally, the weight ratio of 5 : 1 for AM to AS in accord with the ancient preparation showed the best properties of DBT to achieve the maximum activity [14–16]. However, until now, there was no study to assess the antiallergic and anti-inflammatory effect of multiformulas DBT prepared from AM and AS compared with the single extract of AM or AS from the perspective of herb pairs [17].

Based on these backgrounds, we investigated the efficacy and the mechanism of DBT (the weight ratio of 5 : 1 for AM to AS) on allergic and inflammatory skin reaction compared to the single extract of AM or AS via AD-like mouse model.

## 2. Materials and Methods

### 2.1. Preparation of Sample

AM and AS were prepared same as previous reports [18, 19]. Briefly, each of the AM and AS crude materials was extracted with 300 mL of 70% ethanol for 24 h. The extracts were filtered, concentrated, lyophilized, and stored at −80°C. The yield of AM dried extract was approximately 25.0% (w/w, dry weight 7.5 g) and the extract of AS yielded 37.3% (w/w) for dry weight 11.2 g. Each voucher specimen (# AM001 and # AS070) was deposited in the herbarium of the college of pharmacy's laboratory. DBT mixture amounts of AM and AS were weighed according to a ratio of 5 to 1 and then mixed well in a vortex. A voucher specimen of DBT (# DBD E70) was deposited at our laboratory.

### 2.2. Standardization of DBT

**Figure 1:**
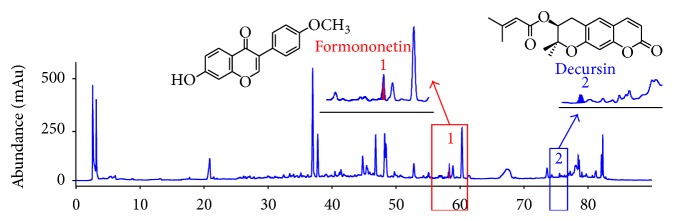
HPLC chromatogram of the DBT by HPLC analysis. The amounts of formononetin and decursin in the DBT extracts were determined as marker chemicals.

DBT was identified by formononetin and decursin using reverse-phase high-performance liquid chromatography (HPLC). 50 mg DBT was mixed with 1 mL methanol, sonicated for 30 min, and filtered through a 0.2 *μ*m filter membrane. HPLC was performed by an Agilent 1100 series instrument and chromatographic separation was achieved on a SHISEIDO CAPCELL PAK C18 column (250 mm × 4.6 mm, 5 *μ*m). Gradient elution was carried out with A : B (water : acetonitrile) as follows: 0 min, 99 : 1; 10 min, 99 : 1; 70 min, 50 : 50; 80 min, 0 : 100; 90 min, 0 : 100. The flow rate was 1.0 mL/min and the detection wavelength was 230 nm. The column temperature was maintained at 40°C. AM and AS were, respectively, characterized based on the content of formononetin and decursin (Figure 1).

### 2.3. Animal Treatment

Six-week-old female BALB/c mice were supplied by Raon Bio (Yongin, Republic of Korea). The mice were maintained in climate-controlled quarters with a 12 h light/12 h dark cycle (at 22–24°C, 55–60% humidity) and provided with access to a standard laboratory diet and water* ad libitum*. After 1 week of adaptation, the mice were randomly divided into six groups of 5 animals each: (1) vehicle: vehicle application, (2) DNCB: 2,4-dinitrochlorobenzene application with vehicle application as a negative control group, (3) DEX: dexamethasone (10 *μ*M/100 *μ*L/day, Sigma Aldrich, MO, USA) treatment with DNCB application as a positive control group, (4) AM: AM (100 mg/mL, 100 *μ*L/day) treatment with DNCB application, (5) AS: AS (20 mg/mL, 100 *μ*L/day) treatment with DNCB application, and (6) DBT: DBT (120 mg/mL, 100 *μ*L/day) treatment with DNCB application.

In brief, the dorsal hair of mice was removed by an electronic hair clipper for sensitive skin. After 24 h, 100 *μ*L of 1% DNCB solution (acetone : olive oil = 4 : 1, v/v solution) was applied on the back skin once a day for 3 d. After 4 days of sensitization, 100 *μ*L of 0.5% DNCB solution was treated on the back during 10 days. 4 h before DNCB application, DEX, AM, AS, and DBT dissolved in phosphate-buffered saline (PBS) were topically applied to the dorsal skin. Before sample treatment, 100 *μ*L of 4% sodium dodecyl sulfate (SDS) was applied to the lesions in order to remove cuticle and to help the absorption of sample [20]. At the end of experiment, serum was obtained by cardiac puncture and the dorsal skin was collected for molecular indicators. All procedures were performed in accordance with the guidelines of the Committee on Care and Use of Laboratory Animals of Kyung Hee University (KHUASP (SE)-14-030).

### 2.4. Histological Observation

To investigate the effects of DBT on DNCB-induced AD-like symptoms in mice, we evaluated the skin thickness. To evaluate skin thickening and mast cell infiltration, the dorsal skin samples (1 × 0.4 cm^2^) were obtained at the end of the experiment (on day 19). The sample was fixed in 10% buffered formalin (Sigma Aldrich, MO, USA) for at least 24 h, progressively dehydrated in solution containing an increasing percentage of ethanol (70%, 80%, 95%, and 100%, v/v), embedded in paraffin under vacuum, and sectioned at 4 *μ*m thickness. Deparaffinized skin sections were stained with hematoxylin and eosin (H&E) for skin thickening and toluidine blue for mast cell infiltration. Histopathological changes were examined using the Leica Application Suite (LAS; Leica Microsystems, Buffalo Grove, IL). The magnification was ×100. The epidermal thickness was measured from the top layer (stratum corneum) to the bottom layer (stratum basale). The dermis thickness was measured in vertical distance between stratum basale layer and the subcutaneous tissues. Thickness was measured 3 times at regular intervals in one slide and obtained 15 results per group [21]. The number of mast cells was measured in the entire area of slides for each sample (*n* = 5).

### 2.5. Measurement of Scratching Behavior

The mice were monitored for 20 min using a digital-camera (model NEX-C3, Sony, Japan), 1 h after last DNCB sensitization. Scratching movement was determined by replaying the recorded video. One incident of scratching was defined as raising to lowering of a leg including a series of scratches at one time.

### 2.6. Measurement of Total Serum Immunoglobulin E (IgE) Levels and Cytokines

The collected blood was centrifuged for 30 min at 16,000 ×g, and serum sample was stored in −80°C until analysis. Serum concentrations of IgE were measured using mouse IgE ELISA kit (BD Pharmingen, CA, USA) according to the manufacturers' instructions. To measure cytokine on dorsal skin changes according to the topical application, the dorsal skin was removed from each mouse (100 mg, *n* = 5 per group) and homogenized. The dorsal skin was lysed using tissue protein extraction reagent (T-PER; Pierce, Rockford, IL, USA) containing a protease inhibitor cocktail (Roche, Indianapolis, IN, USA). The resulting lysate was centrifuged at 16,000 ×g for 30 min at 4°C and stored at −80°C until analysis. Protein concentrations were requantified under identical conditions using a protein assay reagent (Bio-Rad, Hercules, CA, USA).

### 2.7. Preparation of Protein Extraction in Dorsal Skin

Extraction of cytoplasmic and nuclear proteins was performed with standard protocols and our previous paper [22]. In brief, the cytoplasmic buffer (10 mM HEPES, pH 7.9, 10 mM KCl, 0.1 mM EDTA, 0.1 mM EGTA, 1 mM DTT, 0.15% Nonidet P-40, 50 mM *β*-glycerophosphate, 10 mM NaF, and 5 mM Na_3_VO_4_, containing the protease inhibitor cocktail) was used to analyze the phosphorylated I*κ*B*α* in the cytoplasm and nuclear buffer (20 mM HEPES, pH 7.9, 400 mM NaCl, 1 mM EDTA, 1 mM EGTA, 1 mM DTT, 0.50% Nonidet P-40, 50 mM *β*-glycerophosphate, 10 mM NaF, and 5 mM Na_3_VO_4_, containing the protease inhibitor cocktail) was used to analyze the NF-*κ*B protein levels in the nucleus. MAPKs (extracellular signal-regulated kinases; ERK1/2, p38 kinases, the c-Jun N-terminal kinases; JNK) were confirmed by total protein extracts using RIPA assay buffer containing protease inhibitor cocktail.

### 2.8. Detection of Inflammatory Protein Expression

Each denatured protein (30 *μ*g; nuclear, cytoplasmic, and whole fraction) was loaded onto 15% polyacrylamide gels for electrophoresis. Then, the proteins were transferred to polyvinylidene fluoride (PVDF) membranes and incubated at room temperature for 1 h with 5% BSA (diluted in TBS-T; TBS buffer containing 0.1% Tween) to block nonspecific binding. Primary antibodies reactive to mouse *β*-actin (Santa Cruz, USA), phosphorylated NF-*κ*B (Santa Cruz, USA), phosphorylated I*κ*B*α* (Santa Cruz, USA), ERK1/2 (Cell Signaling, USA), phosphorylated ERK1/2 (Cell Signaling, USA), p38 MAPK (Cell Signaling, USA), phosphorylated p38 MAPK (Cell Signaling, USA), JNK (Cell Signaling, USA), and phosphorylated JNK (Cell Signaling, USA) were used overnight (1 : 1,000 dilution; in TBS-T). The membrane was washed three times in TBS-T for 30 min, incubated with horseradish peroxidase-conjugated secondary antibodies (1 : 2,000 dilution; in TBS-T) for 2 h at room temperature (RT), washed three times in TBS-T for 30 min, and revealed with enhanced chemiluminescence (ECL). Immunoreactive bands were detected using an LAS-4000 mini system (Fujifilm Corporation, Tokyo, Kumamoto, Japan).

### 2.9. Statistical Analysis

All data are expressed as the mean ± standard deviation (SD). Significance was determined using one-way ANOVA with Duncan's multiple range test. In all analyses, *P* < 0.05 indicated statistical significance. GraphPad Prism 5 software (San Diego, CA, USA) was used for the statistical analysis.

## 3. Results

### 3.1. Amelioration of Hyperkeratosis and Hyperplasia

**Figure 2:**
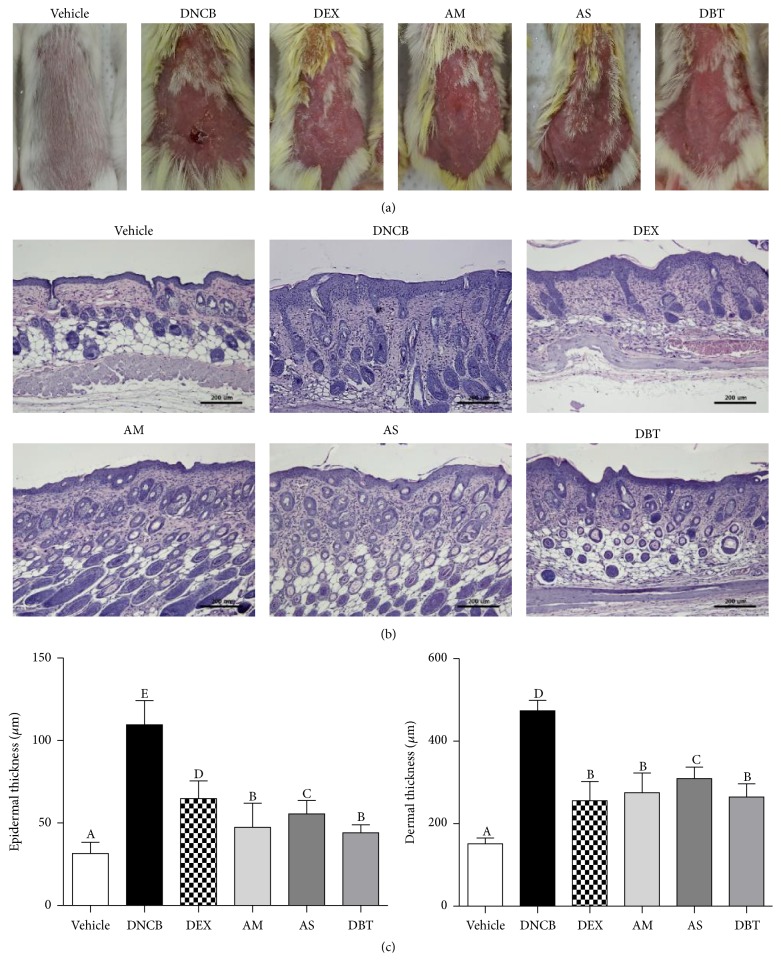
Effects of DBT on histological features of dorsal skin in DNCB-induced AD mice. (a) Representative mice of each treatment group on day 19. (b) Histological observation of the dorsal skin of each group by H&E staining. (c) The thickness of epidermis and dermis. Results are expressed as mean ± SD (*n* = 5). Magnifications are ×100 (scale bar: 200 *μ*m).

AD symptoms including dryness, erythema, and swelling were evidently seen in DNCB group. On the other hand, DBT-treated group significantly reduced AD symptoms (Figure 2(a)). As shown in microscopic analysis (Figure 2(b)), the dorsal skin of DNCB-treated mice (epidermis: 111.6 ± 15.5 *μ*m, dermis: 505.3 ± 31.0 *μ*m) was swollen and significantly thicker than those of the vehicle group (32.3 ± 6.3 *μ*m, dermis: 178.3 ± 31.2 *μ*m). Treatment with AM (epidermis: 43 ± 9.1 *μ*m, dermis: 312.2 ± 51.0 *μ*m), AS (epidermis: 65.8 ± 13.5 *μ*m, dermis: 354.9 ± 49.3 *μ*m), and DBT (epidermis: 39.1 ± 6.9 *μ*m, dermis: 327.8 ± 38.2 *μ*m) markedly attenuated DNCB-induced hyperkeratosis and hyperplasia. Particularly, the epidermis and dermis thickness of DBT-treated group were lower than those in AS-treated group.

### 3.2. Attenuation of Scratching Behavior

**Figure 3:**
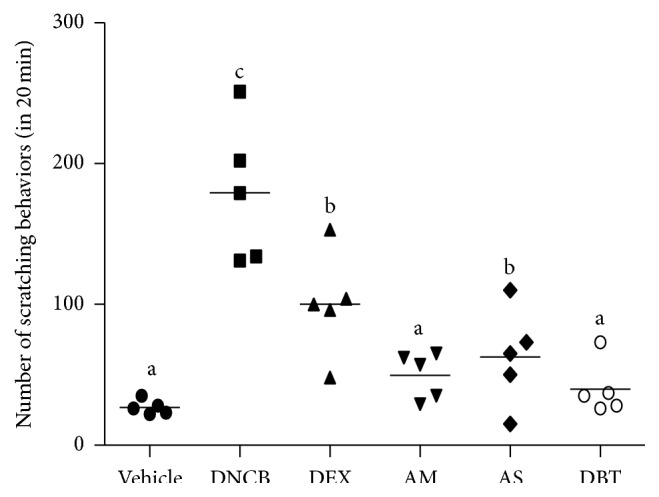
Effects of DBT on scratching behavior. The numbers of scratching behaviors are expressed as mean ± SD (*n* = 5).

Intensive pruritus leads to extensive scratching as a hallmark of AD [23]. The distribution of the assessed level of scratching behavior was illustrated as the dot plot (Figure 3). The scratching behavior was markedly increased in DNCB-treated mice (179 ± 50) compared with the vehicle group (27 ± 5). This increased scratching behavior was significantly reduced by AM (50 ± 16), AS (63 ± 35), and DBT (40 ± 19) treatment. Consistently with histologic analysis, the reduction of pruritus by DBT treatment was greater than AS single treatment.

### 3.3. Inhibition of the Number of Mast Cells

**Figure 4:**
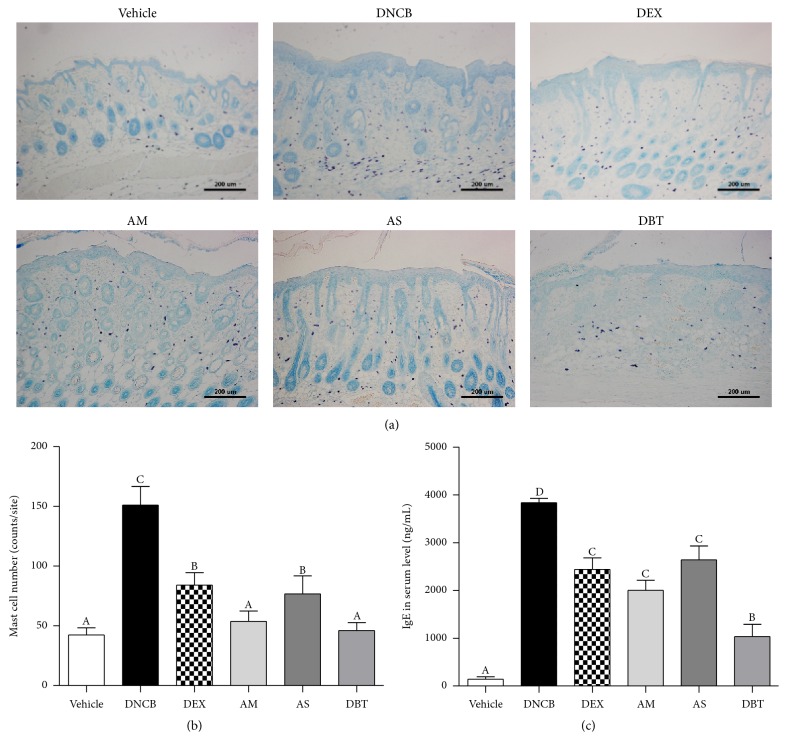
The number of mast cells and the level of IgE in DNCB-induced AD mice. (a, b) The number of mast cells observed by toluidine blue staining. Magnifications are ×100 (scale bar: 200 *μ*m). (c) The level of IgE in serum. Results are expressed as mean ± SD (*n* = 5).

The number of toluidine blue-stained mast cells of DNCB-treated mice (151 ± 15) was significantly increased compared with that of the vehicle group (44 ± 6). AM (56 ± 9), AS (70 ± 12), and DBT (45 ± 6) markedly lowered the number of mast cells in the skin of DNCB-treated mice (Figures 4(a) and 4(b)). Compared with AS, DBT significantly decreased the number of mast cells.

### 3.4. Reduction of Serum IgE Level

We investigated whether DBT alters the serum level of IgE in DNCB-induced AD-like mice. We found that the level of IgE was markedly increased by DNCB application, compared to vehicle group. Treatment with AM, AS, DBT, and DEX group significantly reduced the IgE level of DNCB group (Figure 4(c)). DBT treatment showed more effective reduction in IgE level than the single extract of AM or AS.

### 3.5. Downregulation of Cytokines

**Figure 5:**
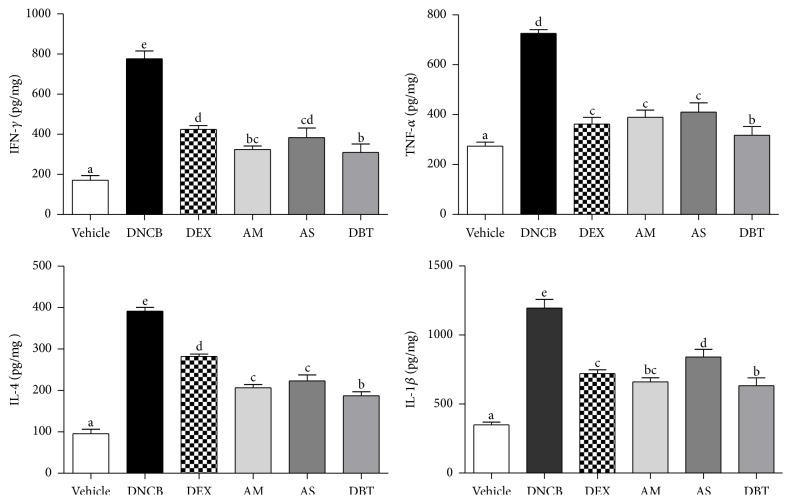
Effects of DBT on levels of Th2- and Th1-type cytokines. Values of cytokines are means ± standard error of the mean.

Lesional skin of AD patients exhibits increased expression of Th2, Th1 cytokines and proinflammatory cytokines [24]. The cytokine levels in dorsal skin were significantly increased in DNCB (IL-4: 454.9%, IL-6: 1339.7%, IFN-*γ*: 524.6%, TNF-*α*: 247.8%, and IL-1*β*: 342.0%) compared to vehicle. Topical treatment of DEX, AM, AS, and DBT showed significantly lower levels of various cytokines compared with DNCB group (Figure 5). Particularly, the levels of IL-4 and TNF-*α* in DBT are significantly lower than in the single extract of AM or AS.

### 3.6. Inhibition of Inflammatory Mediators

**Figure 6:**
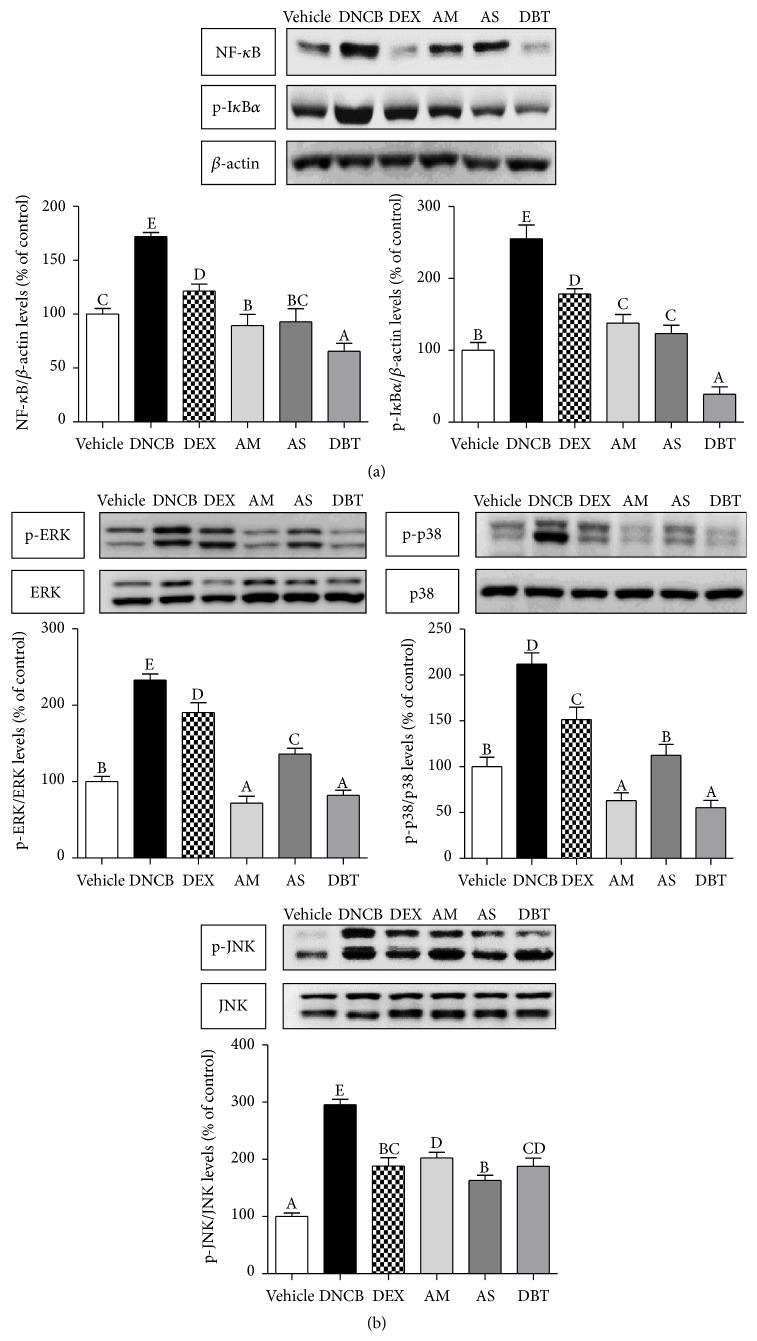
Effects of DBT on inflammatory mediators in DNCB-induced AD mice. (a) NF-*κ*B and phosphor-I*κ*B*α* levels (b) phosphor-MAPKs (ERK1/2, p38, and JNK) levels. Results are expressed as mean ± SD (*n* = 5).

Western blot analysis showed that DNCB challenge markedly upregulated the NF-*κ*B (about 1.7-fold) and phosphorylation of I*κ*B*α* (about 2.6-fold) compared to the vehicle group, whereas simultaneous treatment with DEX, AM, AS, and DBT attenuated the DNCB-induced NF-*κ*B activation and phosphorylation of I*κ*B*α* (Figure 6(a)). Moreover, DBT significantly reduced NF-*κ*B and phosphor-I*κ*B*α* expressions as compared with the AM and AS groups. MAPKs phosphorylation was significantly upregulated by DNCB, including phosphorylation ERK, p38, and JNK pathways (Figure 6(b)). AM, AS, and DBT treatment inhibited the increased level of phosphorylation of ERK, p38, and JNK. The regulation of phospho-ERK and phospho-p38 levels in DBT was more effective than AS, whereas phospho-JNK level was even higher than that of AS.

## 4. Discussion

Hyperplasia is one of the main symptoms in AD [24]. In histological analysis, we confirmed that the dermis and epidermis were thickened in the DNCB-induced group compared to vehicle group. Our findings showed that thickening of the epidermis and dermis was significantly reduced in DBT, AS, and AM groups. These findings are in agreement with those of a previous study presenting that DBT significantly inhibited ear swelling compared with DNCB-sensitized mice [12]. Particularly, topical application of DBT markedly suppressed a skin thickening and hyperkeratosis of the epidermis as compared with the AS groups. Scratching behavior in DNCB-induced model could be a major feature in skin lesions as results of various immunological responses such as the elevation of serum IgE concentration and number of mast cells [1, 22]. Therefore, it is important to decrease the scratching behavior in controlling the skin lesions and various immunological reactions. DBT significantly inhibited the itching sign and decreased the elevation of serum IgE levels and degranulation of mast cells (MCs) compared to DNCB-induced group. These results are in close agreement with the findings of a similar previous study [12]. Particularly, DBT was more significantly effective on the inhibition of serum IgE levels compared to the single extract of AM or AS. These improvements could be partially attributed the antiallergic properties of DBT compared to the single extract of AM or AS in AD.

AD is involved in the dysregulation of Th type 1 and 2 cell-mediated immune responses [2]. Th2 cytokines, such as IL-4 and IL-6, promote B cell proliferation and cause IgE class switching in both acute and chronic AD [25]. On the other hand, Th1 cytokines, such as IFN-*γ* and IL-1*β*, are upregulated mainly in chronic stage [26]. Specifically, TNF-*α* regulates dermal-epidermal interactions in keratinocyte during inflammation, wound healing, and epidermal growth. Also, TNF-*α* stimulates IL-6 with enhancing the IL-4-induced IgE production [18, 22]. In close accordance with a previous study [12], topical application of DBT significantly inhibited the expression of AD-related pathogenic cytokines such as IL-4, IL-6, IFN-*γ*, TNF-*α*, and IL-1*β* regardless of Th1 and Th2 cytokines similarly to DEX group. Furthermore, DBT significantly suppressed DNCB-induced elevation of IL-4 and TNF-*α* compared to the single extract of AM or AS. Our comprehensive findings indicate that DBT may be more effective on suppressing an immune response by inhibiting both Th1- and Th2-type cytokine production as compared with the single extract of AM or AS in AD.

NF-*κ*B signaling pathway, which is mediated by TNF-*α*, plays a critical role in the cellular immune and inflammatory response in epidermal keratinocytes [27]. In this study, we confirmed that activation of NF-*κ*B results in skin thickening and that DBT reduced hyperplasia of epidermis in mice by suppressing expression of NF-*κ*B. In addition, we have observed that the translocation of NF-*κ*B to nucleus by DBT was inhibited as shown by a marked decrease of NF-*κ*B in the nucleus and a decrease of phosphorylated I*κ*B*α* in the cytoplasm.

Mitogen-activated protein kinase (MAPK) signaling is important in inflammatory skin diseases by controlling the activation, proliferation, degranulation, and migration of various immune cells. MAPK is divided into three groups: ERK controlling cell cycle progression, JNK regulating the cell proliferation and survival, and p38 MAPK relating to cell growth and differentiation, cell death, and inflammation [28]. Several studies have suggested that the development of MAPK inhibitors could be a therapeutic target for allergic diseases [29]. In the present study, DNCB challenge induced the increased activities of MAPK in consistency with the results of previous studies. Topical application of DBT inhibited the phosphorylation of ERK and p38 more than AS groups. These data suggest that inhibition of MAPK by DBT may contribute to its antiallergic and anti-inflammatory activities.

In conclusion, DBT with the weight ratio of 5 : 1 for AM to AS in accord with the ancient preparation not only prevented the degranulation of MCs and regulated the NF-*κ*B signaling pathway, but also suppressed the phosphorylation of MAPK signaling molecules. Particularly, DBT formula could be more effective than AM or AS single treated groups on the antiallergic reactions by suppressing NF-*κ*B signaling pathway and Th2-type cytokines mediating by TNF-*α*. These consecutive antiallergic and anti-inflammatory effects of DBT are believed to inhibit epidermal and dermal thickness and scratching behavior and to contribute significantly to the clinical efficacy in the management of AD.
